# Imaging of calcific tendinopathy around the shoulder: usual and unusual presentations and common pitfalls

**DOI:** 10.1007/s11547-020-01300-0

**Published:** 2020-11-05

**Authors:** Domenico Albano, Alessandra Coppola, Salvatore Gitto, Santi Rapisarda, Carmelo Messina, Luca Maria Sconfienza

**Affiliations:** 1 IRCCS Istituto Ortopedico Galeazzi, Unità Operativa di Radiologia Diagnostica ed Interventistica, Via Riccardo Galeazzi 4, 20161 Milan, Italy; 2 Sezione di Scienze Radiologiche, Dipartimento di Biomedicina, Neuroscienze e Diagnostica Avanzata, Università degli Studi di Palermo, Via del Vespro 127, 90127 Palermo, Italy; 3 Scuola di Specializzazione in Radiodiagnostica, Università degli Studi di Milano, 20122 Milan, Italy; 4 Dipartimento di Scienze Biomediche per la Salute, Università degli Studi di Milano, Via Pascal 36, 20133 Milan, Italy

**Keywords:** Calcific tendinopathy, Rotator cuff, Ultrasound, Conventional radiography, Magnetic resonance, Pitfall

## Abstract

Rotator cuff calcific tendinopathy (RCCT) is a very common condition, characterized by calcium deposition over fibrocartilaginous metaplasia of tenocytes, mainly occurring in the supraspinatus tendon. RCCT has a typical imaging presentation: in most cases, calcific deposits appear as a dense opacity around the humeral head on conventional radiography, as hyperechoic foci with or without acoustic shadow at ultrasound and as a signal void at magnetic resonance imaging. However, radiologists have to keep in mind the possible unusual presentations of RCCT and the key imaging features to correctly differentiate RCCT from other RC conditions, such as calcific enthesopathy or RC tears. Other presentations of RCCT to be considered are intrabursal, intraosseous, and intramuscular migration of calcific deposits that may mimic infectious processes or malignancies. While intrabursal and intraosseous migration are quite common, intramuscular migration is an unusual evolution of RCCT. It is important also to know atypical regions affected by calcific tendinopathy as biceps brachii, pectoralis major, and deltoid tendons. Unusual presentations of RCCT may lead to diagnostic challenge and mistakes. The aim of this review is to illustrate the usual and unusual imaging findings of RCCT that radiologists should know to reach the correct diagnosis and to exclude other entities with the purpose of preventing further unnecessary imaging examinations or interventional procedures.

## Introduction

Rotator cuff calcific tendinopathy (RCCT) is a very common condition, characterized by calcium deposition over fibrocartilaginous metaplasia of tenocytes, mainly occurring in the supraspinatus tendon [1–3]. The prevalence of RCCT has been reported to range from 2.7% to 10.3% [4–7]; ∼50% of these patients eventually become symptomatic [8].

The cause of RCCT is not completely understood. It is likely related to a low oxygen tension inside the tendon, although hormonal status may play a role, since RCCT is far more common in women before menopause [1]. As previously described by Uhthoff et al., RCCT is a cell-mediated process with three well-defined phases [9]: (i) *precalcific stage,* with fibrocartilaginous tendon transformation; (ii) *calcific stage,* with calcium crystals deposition (formative phase) followed by their resorption due macrophages activation (resorptive phase) [10]. During this stage, edema, and extravasation of calcium crystals in the subacromial bursa occur, leading to increased intratendinous pressure and pain; (iii) *postcalcific stage,* with tendon matrix remodeling by fibroblasts and replacement of calcium crystals by granulation tissue, leading to complete tendon healing. In the pre- and post-calcific stages, RCCT may be totally asymptomatic or cause mild pain; conversely, in the resorptive phase, RCCT can be a non-negligible cause of intense shoulder pain, generally not responding to common oral painkillers or anti-inflammatory drugs, and often leading patients to seek for emergency medical consultation [1–3].

Several treatments for RCCT are currently in use, including the use of nonsteroidal anti-inflammatory drugs as first approach to relieve pain in the acute phases, physiotherapy to prevent articular stiffness, bursal steroid injections, ultrasound (US)-guided percutaneous irrigation of calcific tendinopathy (US-PICT) and extracorporeal shock wave therapy (ESWT) [11–19]. Surgery is recommended only when conservative treatment is unsuccessful [13].

Imaging is crucial in the differential diagnosis of RCCT, being nodular radiopaque deposits at conventional radiography (CR) the most common presentation. However, the different stages on this condition and the unusual location where calcific deposits may migrate could make the final diagnosis quite tricky. Thus, our aim is to present the usual and unusual imaging findings of RCCT to avoid some of the most common mistakes when this condition does not have a typical presentation.

## Usual imaging findings of RCCT

### Conventional radiography

RCCT typically presents with calcific deposits around the humeral head involving the RC tendons, specifically the supraspinatus tendon (80% of cases), the lower side of the infraspinatus tendon (15% of cases), and the preinsertional part of the subscapularis tendon (5% of cases) [3, 20].

**Fig. 1:**
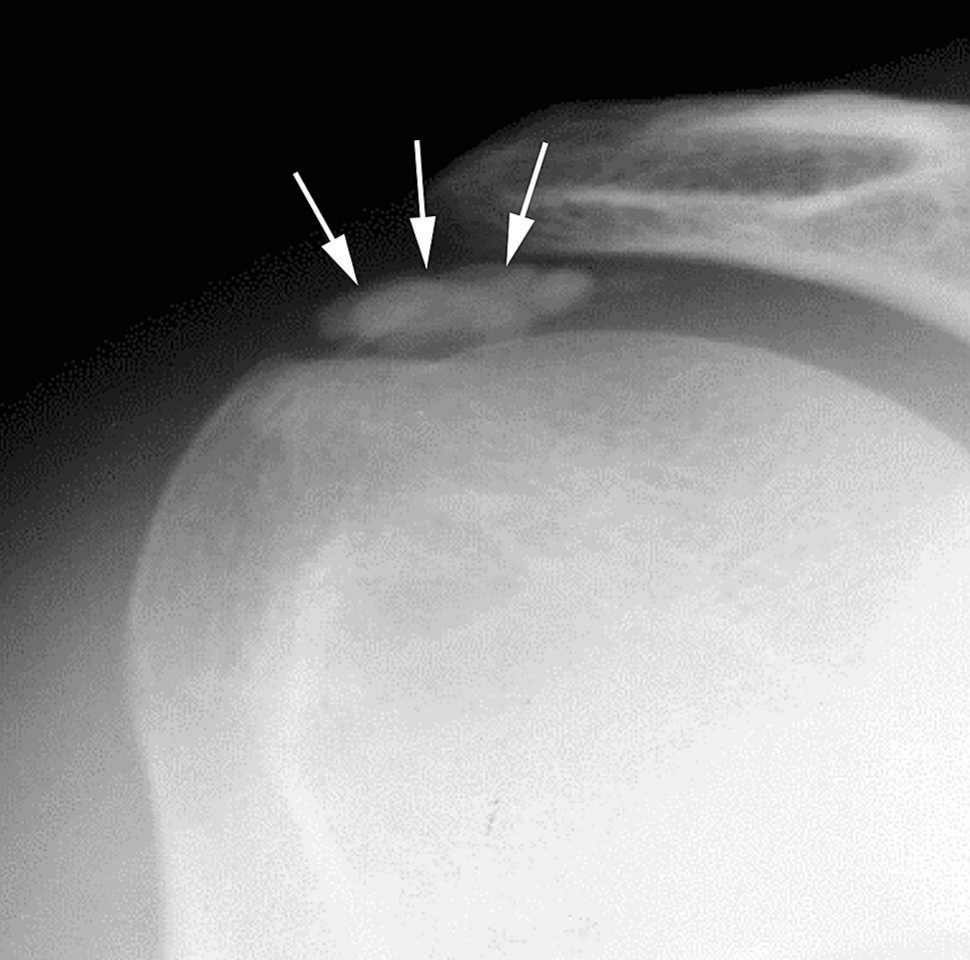
Right shoulder antero-posterior plain radiography of a 35-year-old female patient with painful RCCT showing an opacity without trabeculation over the humeral head (arrows)

CR and US are generally the preferred methods to diagnose RCCT [21, 22]. CR usually represents the first imaging modality performed in patients complaining of shoulder pain and the first examination used when RCCT is suspected, especially when presenting in the emergency room with untreatable pain [13]. Standard shoulder CR generally includes anteroposterior view in internal and external rotations, although the additional projection of axillary lateral view can be helpful to detect subscapularis tendon calcification [2]. In their usual manifestations, calcifications appear as homogeneous and amorphous opacities with smooth or ill-defined margins, without trabeculation (Fig. 1) [23].

Several radiographic classifications based on size or morphological features have been proposed, but none of them has shown sufficient reliability and reproducibility [13]. The classification by Gartner and Heyer seems to be the most useful in clinical practice, as it correlates with the histological stage: (i) well circumscribed and dense calcification; (ii) soft contour/dense or sharp transparent; and (iii) translucent and cloudy appearance without clear circumscription [20].

Deposits are well visible during calcific stage (i and ii) while in the resorptive phase are barely visible on CR (iii).

### Ultrasound

**Fig. 2:**
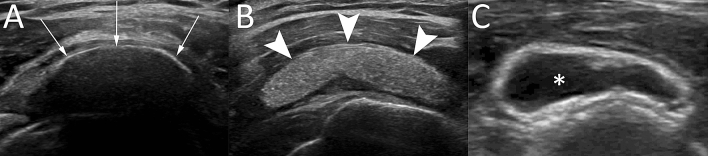
Shoulder US of three different patients with painful RCCT. (**A**) A hard calcification, with hyperechoic rim and strong posterior acoustic shadow (arrows). (**B**) A soft calcification, appearing as homogeneously hyperechoic without posterior acoustic shadow (headarrows). (**C**) A fluid calcification, presenting a thin peripheral hyperechoic rim and an anechoic core (asterisk)

**Fig. 3:**
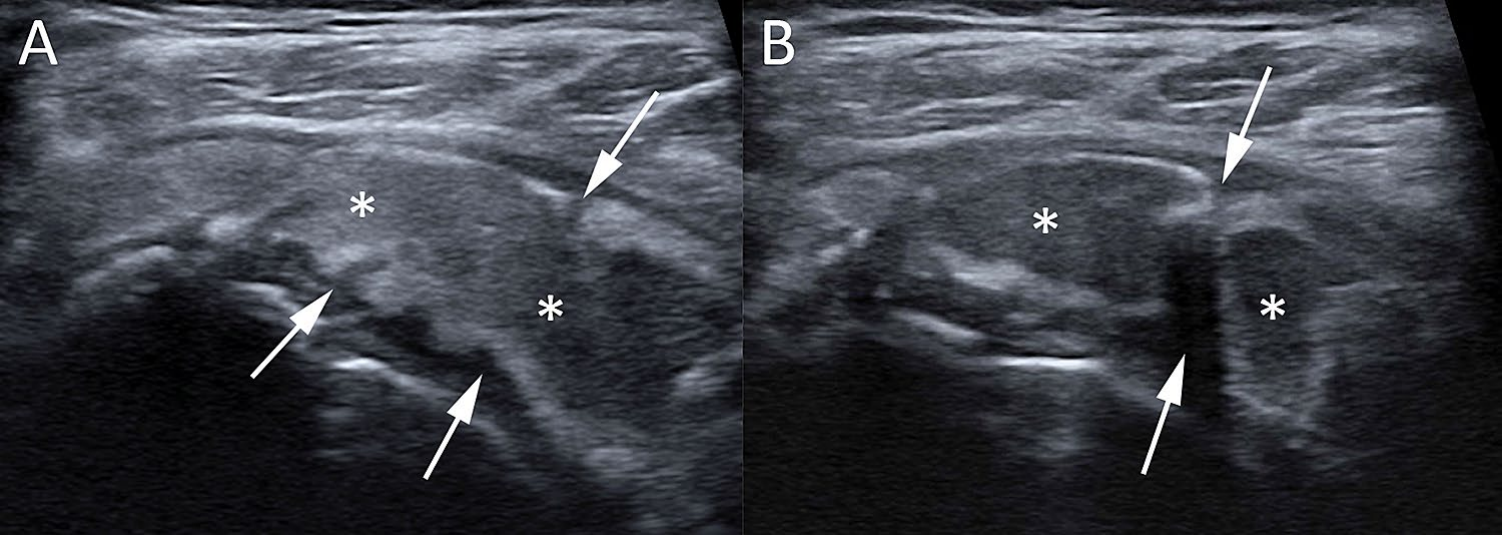
Shoulder US of a 52-year-old male patient with recent onset of acute pain that was unresponsive to analgesics. The US (**A**, **B**) shows a large calcification in the resorptive phase presenting irregular profiles and focal breaks (arrows) with iso-hypoechoic fluid content (asterisks)

US has proved to be a good diagnostic tool for detecting and localizing calcifications within the RC tendons, with 98% sensitivity and 94% specificity [5, 24]. Calcific deposits typically appear as hyperechoic foci with well-defined acoustic posterior shadowing but, due to the variable of calcific content, sometimes they can appear as hyperechoic clusters with faint or absent acoustic shadow [25]. Several classifications have been proposed based on calcium amount and US morphology. Sconfienza et al. proposed an US classification related to the three different types of RCCT commonly encountered in patients undergoing interventional procedures: (i) hard calcifications, with hyperechoic rim and strong posterior acoustic shadow (Fig. 2A); (ii) soft calcifications, when appearing as homogeneously hyperechoic, almost isoechoic to the normal tendon, without posterior acoustic shadow (Fig. 2B); and (iii) fluid calcifications, when presenting with a thin peripheral hyperechoic rim and a hypoechoic or anechoic core (Fig. 2C) [26]. However, calcification appearance can change in the resorptive phase, first showing irregular profiles and focal breaks, then evolving in complete fragmentation of the deposit (Fig. 3).

### Magnetic resonance

**Fig. 4:**
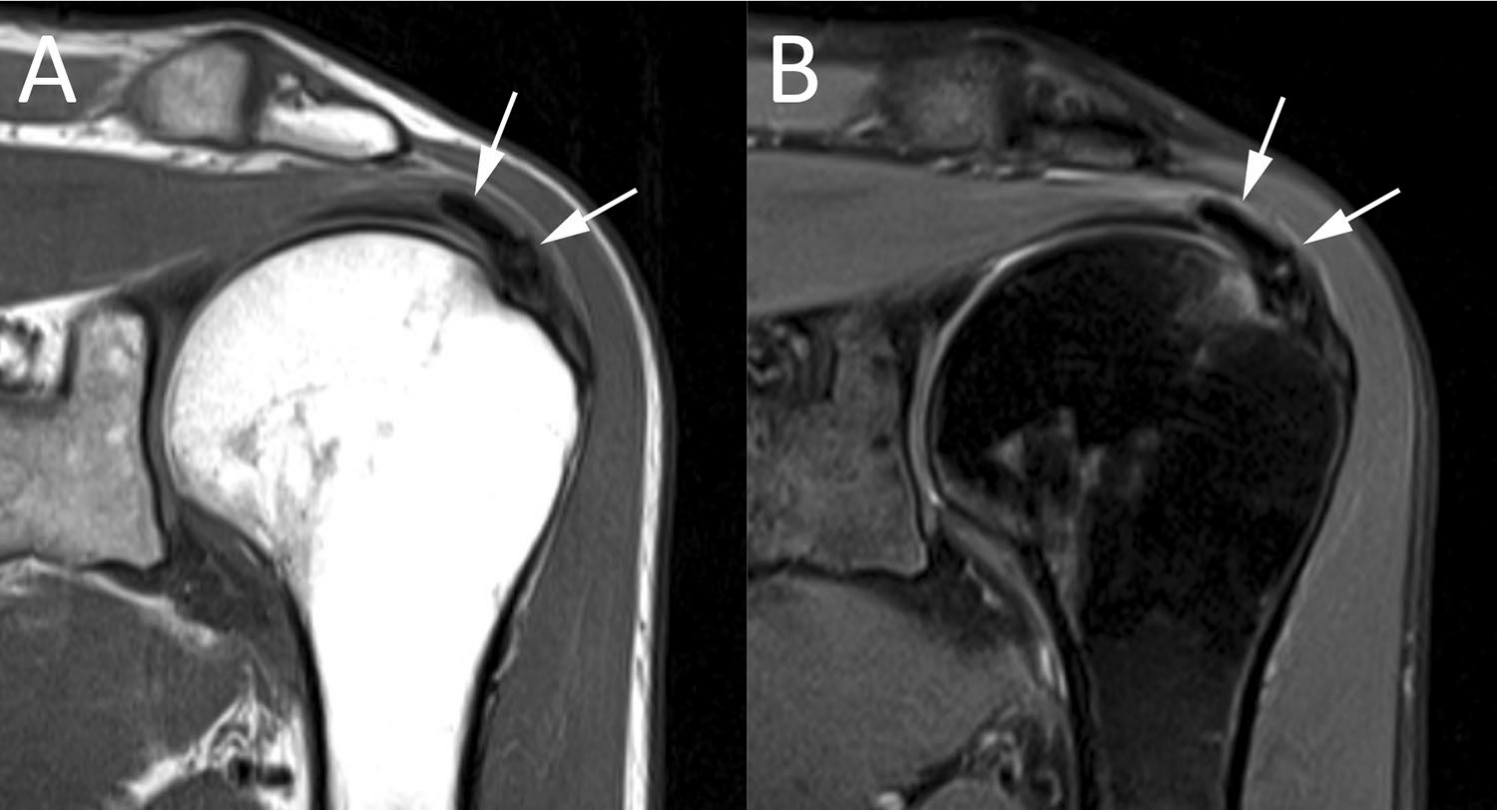
Left shoulder MR of a 47-year-old female patient with painful RCCT. Coronal T1-weighted (**A**) and fat-suppressed proton-density weighted (**B**) images show a calcification appearing as focal area of low signal on all sequences (arrows) within the supraspinatus tendon

Magnetic resonance (MR) is widely performed for shoulder pain and particularly to evaluate the RC disorders [27, 28], although its accuracy in RCCT remains limited (65% sensitivity and 58% specificity), especially being inaccurate for size evaluation, despite its role in determining acuity of the finding [29–32]. On MR, calcifications typically appear as focal areas of low signal on all pulse sequences within the RC tendon (Fig. 4) [33, 34]. On T1-weighted images, calcifications can be categorized similarly to common radiographic classifications, differentiating the shape and density of the calcium deposits and their outline in the tendon structure [30]. Other authors evaluated the diagnostic performance of susceptibility-weighted imaging (SWI) for the detection of shoulder calcific deposits showing 98% sensitivity and 96% specificity compared to CR [31]. Studies on the role of MR arthrography for calcific tendinopathy showed it is insufficient in the diagnosis, as small calcific deposits may be difficult to detect, leading to false-negative, as well as normal hypointense areas within RC tendons may lead to false-positive results [32].

### Common evolution of the disease

**Fig. 5:**
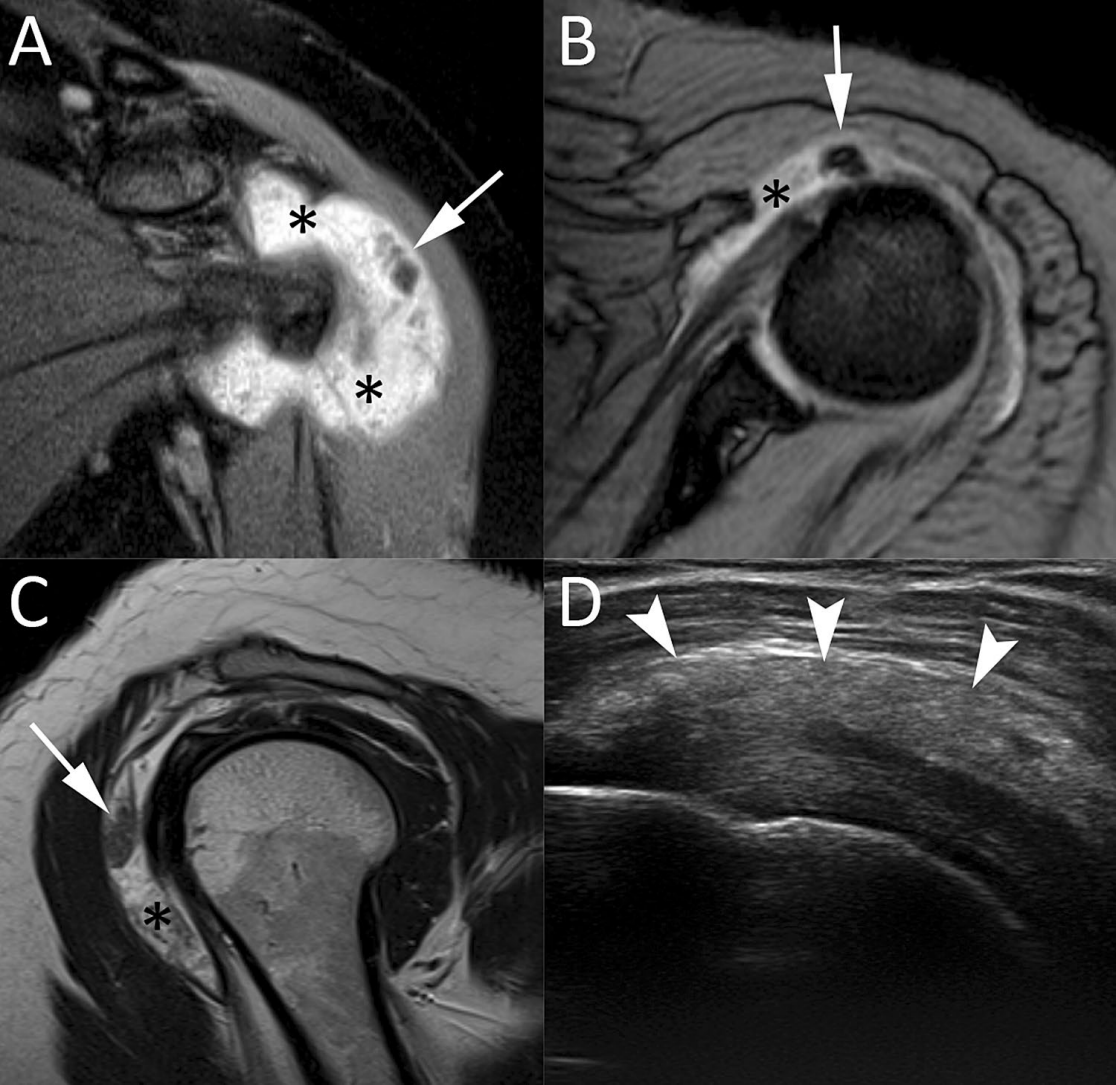
Two cases of bursal migration of RCCT. Coronal fat-suppressed proton-density weighted (**A**), axial gradient-echo (**B**), and sagittal T2-weighted (**C**) MR images of a 26-year-old female patient with atraumatic shoulder pain shows intrabursal migration of a calcification (arrows) with acute bursitis characterized by effusion and synovial hypertrophy within the subacromial–subdeltoid bursa (asterisks). Right shoulder US of a 32-year-old female patient with atraumatic pain (**D**) showing the subacromial–subdeltoid filled with hyperechoic fluid containing calcium and debris (headarrows) over an intact supraspinatus tendon

The resorptive phase of RCCT is characterized clinically by acute pain, occasionally associated to fragmentation and migration of calcium in the surrounding tissues, bursae, or bone. The intra-bursal migration of calcifications can be seen as a dense crescent streak overlying the originating tendon [25, 35, 36]. During migration, intratendinous calcifications are extruded from the tendon into the sub-bursal space and intra-subacromial bursa more frequently [37]. In case of intrabursal migration, CR generally shows a convex calcific layer, fluctuating between the humeral head and the acromion. At US examination, the subacromial bursa appears thickened and filled with inhomogeneous hyperechoic fluid containing calcium and debris, in association with edematous changes in the surrounding fatty space [25, 37]. MR may demonstrate the same findings of US, also showing the presence of associated findings, such as bone edema or joint effusion (Fig. 5) [37].

**Fig. 6:**
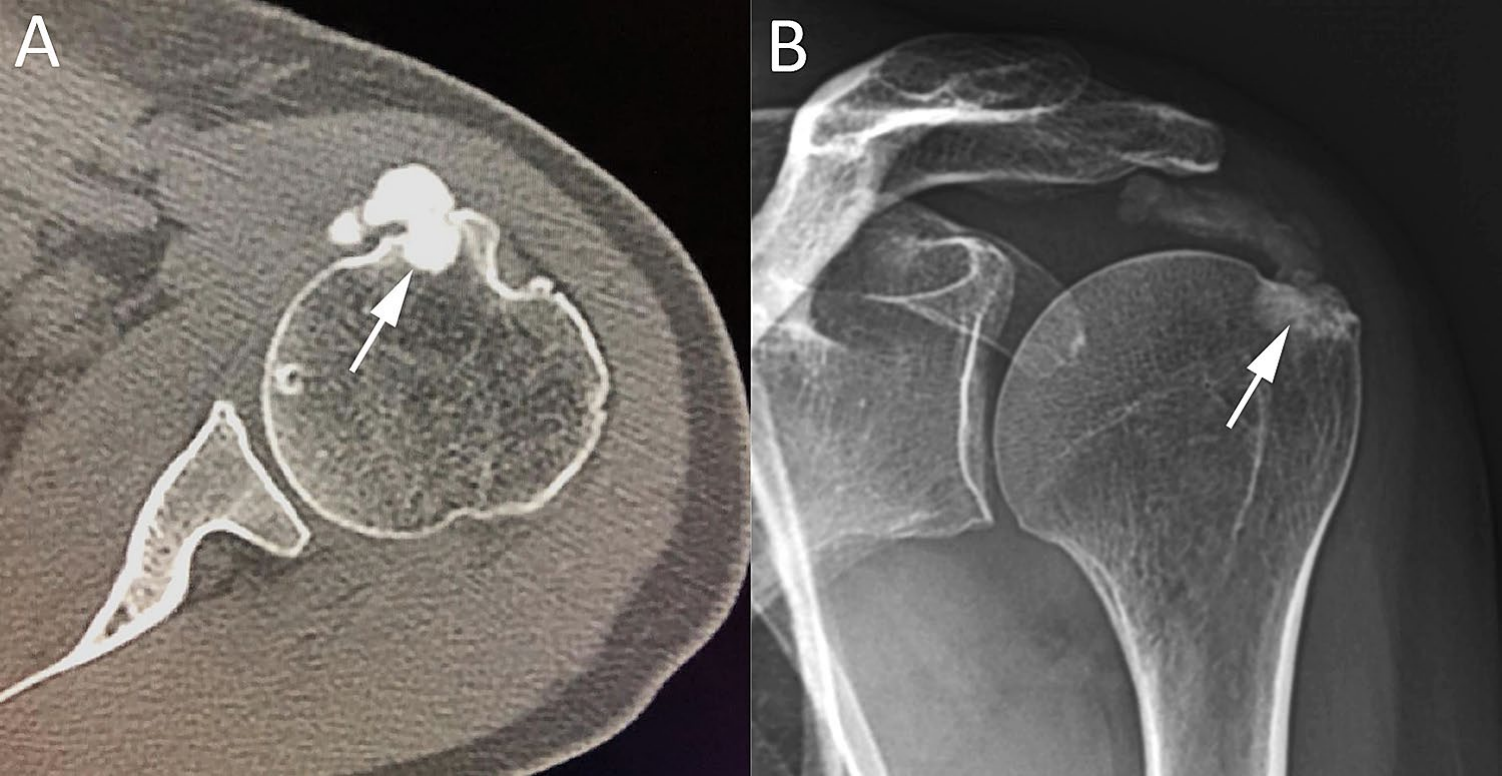
Intraosseous migration of RCCT. Axial shoulder-computed tomography image (**A**) of the left shoulder of a 45-year-old male patient with a large calcification of the subscapularis tendon, which has penetrated the cortical bone of the lesser humeral tuberosity (arrow). Antero-posterior shoulder CR (**B**) of a 54-year-old female patient with intraosseous migration of a large calcification of the supraspinatus tendon (arrow)

RCCT can also appear with aggressive imaging features. In the acute phase, RCCT can present with intraosseous migration of calcific deposits, which induces osseous changes with aggressive features, such as cortical erosion, periosteal reaction, subcortical calcium migration, intramedullary edema, and clearly presence of soft tissue calcification [34, 38]. Intraosseous penetration of calcium from RCCT has been reported in literature by small case series [34, 38–41]. Some of these studies described atypical findings that can mimic malignancy at CR. Usually, it presents as a single sclerotic lesion with radiolucent halo in the humeral head or well-circumscribed sclerosis at the greater tuberosity, associated with cortical erosion and narrow zone of transition [40, 41]. In these cases, it should be considered different diagnostic hypothesis as bone island, osteoid osteoma, infection, and osteoblastic metastases [42]. MR can show an extensive pattern of edema in the perilesional bone marrow mimicking malignancy or extraosseous signal abnormality as focal rim of T1-hypointensity and T2-hyperintensity mimicking infection [40, 41]. Sometimes MR can detect acute tendinopathy or subtle multifocal intrinsic low signal corresponding to the microcalcific deposits that can be helpful in the correct interpretation of images [40]. Mistakes are particularly common on MR, when calcifications are poorly visible [43]. In this case, US can be particularly useful when calcifications are no longer dense enough to be visible on CR for establishing a diagnosis of RCCT (Fig. 6) [38]. Factors that aid to distinguish RCCT from more aggressive entities are the absence of joint effusion or soft-tissue mass. Also, the acute clinical presentation should help orienting the diagnosis [34]. Clinical and imaging follow-up can take advantage of the self-limiting nature of this process and may show its progressive resolution, excluding neoplastic or infectious processes [44].

Another point that should be considered when dealing with this condition is the common evolution of calcific deposits after treatment. As mentioned above, US-PICT and ESWT are the most commonly used procedures to treat RCCT. Previous studies have used CR and/or US to assess imaging response to treatment [45]. Most studies demonstrated a progressive and complete resorption of calcific deposits, while a minor part of authors reported the fragmentation or significant reduction of calcium deposit size already after 1 week [46], 3 months [47], and 12 months [48, 49]. Notably, total calcium resorption has been reported to be higher after US-PICT than ESWT [48, 49]. Furthermore, pre-treatment calcification size seems to be an independent predictor for changes in imaging outcomes after ESWT [45].

As mentioned, RCCT has an almost typical presentation: in most cases, calcific deposits appear as a dense opacity around the humeral head on CR, as hyperechoic foci with acoustic shadow at US, and as a signal void at MR. However, there are common pitfalls and unusual presentations in which RCCT may have a different imaging appearance, thus representing a non-negligible diagnostic challenge.

## Common pitfalls

### Calcific enthesopathy

**Fig. 7:**
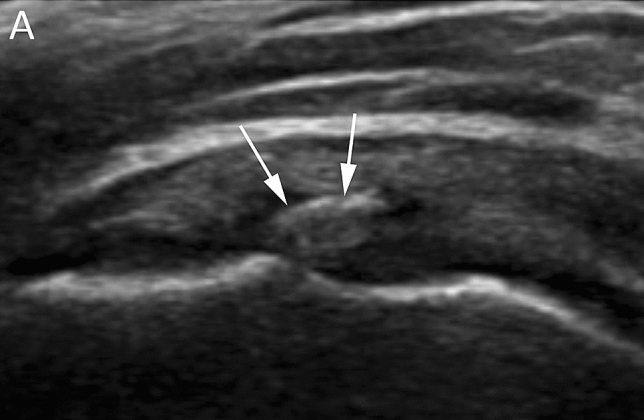
Right shoulder US of a 62-year-old male patient with calcific enthesopathy. US image (**A**) shows a small lamellar hyperechoic area at the insertion of the supraspinatus tendon (arrows)

Calcific enthesopathy represents a common pitfall in the evaluation of RC tendons [50, 51]. This condition is the result of a degenerative process, which appears more often with aging and can be idiopathic or associated with seronegative arthropathies or chronic traction injuries [52]. Occasionally, in calcific enthesopathy, deposition of calcium pyrophosphate may be detected. As a result of a degenerative process, on necrotic areas of tendon, imaging can detect the presence of tiny, lamellar calcifications (Fig. 7) [53] seen at the insertion of the tendon to the bone (enthesis). Enthesopathy does not show resolution and can progress to ossification [50] or to cortical erosions of the bone around the enthesis [51]. This process is totally different from that of RCCT, which is a condition of metaplasia occurring in normal tendons, with the apposition of hydroxyapatite on calcific deposits. Also, after the calcification has completely resorpted, the tendon has a complete *restitutio ad integrum*, an event that never happens in degenerative enthesopathy.

### Mimickers of tendon tear at MR

A possible MR pitfall in shoulder imaging is RCCT that resembles a tendon tear, given that these two conditions may have similar appearance when RCCT occurs in the resorptive phase. Indeed, MR and MR arthrography are considered scarcely adequate in the assessment of RCCT [29, 30, 32, 54], which is generally identified and confirmed by CR, with US having the advantage to recognize the actual location and extent of the calcification itself, as well as associated findings such as subacromial bursitis, RC tears, and biceps tendon pathology [55–58]. In some cases, MR can be performed to exclude other causes of shoulder pain or requested by the orthopedic surgeon for preoperative planning [59].

**Fig. 8:**
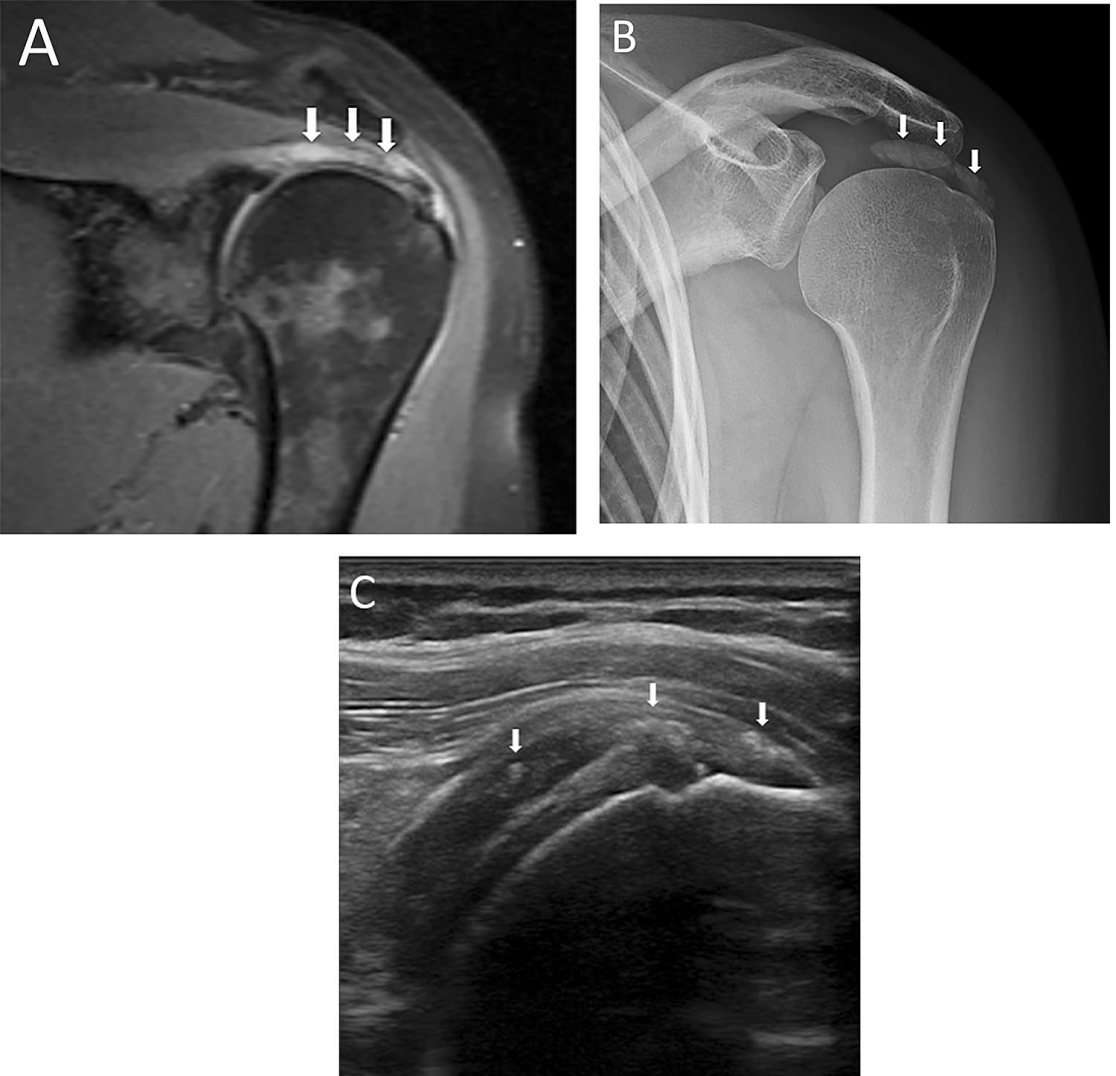
A 47-year-old female patient with intense left shoulder pain resistant to oral anti-inflammatory drugs and no history of trauma. Oblique coronal fat saturated proton density-weighted MR image (**A**) performed 1 week after pain onset shows a focal area of hyperintensity within the supraspinatus tendon without fiber retraction, that was described as partial thickness bursal side tear (arrows). Anteroposterior CR of the left shoulder (**B**) performed 3 days later reveals a large calcification over the humeral head (arrows). The patient underwent shoulder US (**C**) three weeks later, with long-axis US image of supraspinatus tendon showing fragmented hyperechoic calcification with faint acoustic backs shadow (arrows). Notably, US also showed no evidence of supraspinatus tear

However, during the acute phase, RCCT appears differently than the typical focal area of low signal on all pulse sequences [2]. During resorptive phase, deposits are barely visible on CR [23], as the amount of calcium contained in the deposit is low. Siegal et al. explained that fluid sensitive MR sequences can show surrounding areas of increased signal intensity compatible with edema, particularly during the resorptive phase [2]. Merolla et al. specifies that areas of increased signal intensity can be found around deposits in T2-weighted images, corresponding to edema around the deposits in the resorptive phase [60]. This can lead to a misinterpretation of a calcific tendinopathy as a RC lesion. Indeed, areas of increased signal intensity are not located only around the calcification, but also within the calcification itself, which can present with a remarkable edematous component. This could be explained by the macrophages phagocytosis and the development of vascular weaving during the resorptive phase. In this phase, there is a remarkable recalling of water, which is the reason of the consistent signal hyperintensity detected on fluid sensitive images. As RC tears mostly present as hyperintense signal areas corresponding to fluid signal within the tendons on these sequences [61], we suggest that MR should be always assessed in presence of a previous CR or US examination in patients in whom RCCT is suspected. A case of acute RCCT mistaken for a tendon tear at MR is shown in Fig. 8.

## Unusual presentation of RCCT

### Intramuscular migration

**Fig. 9:**
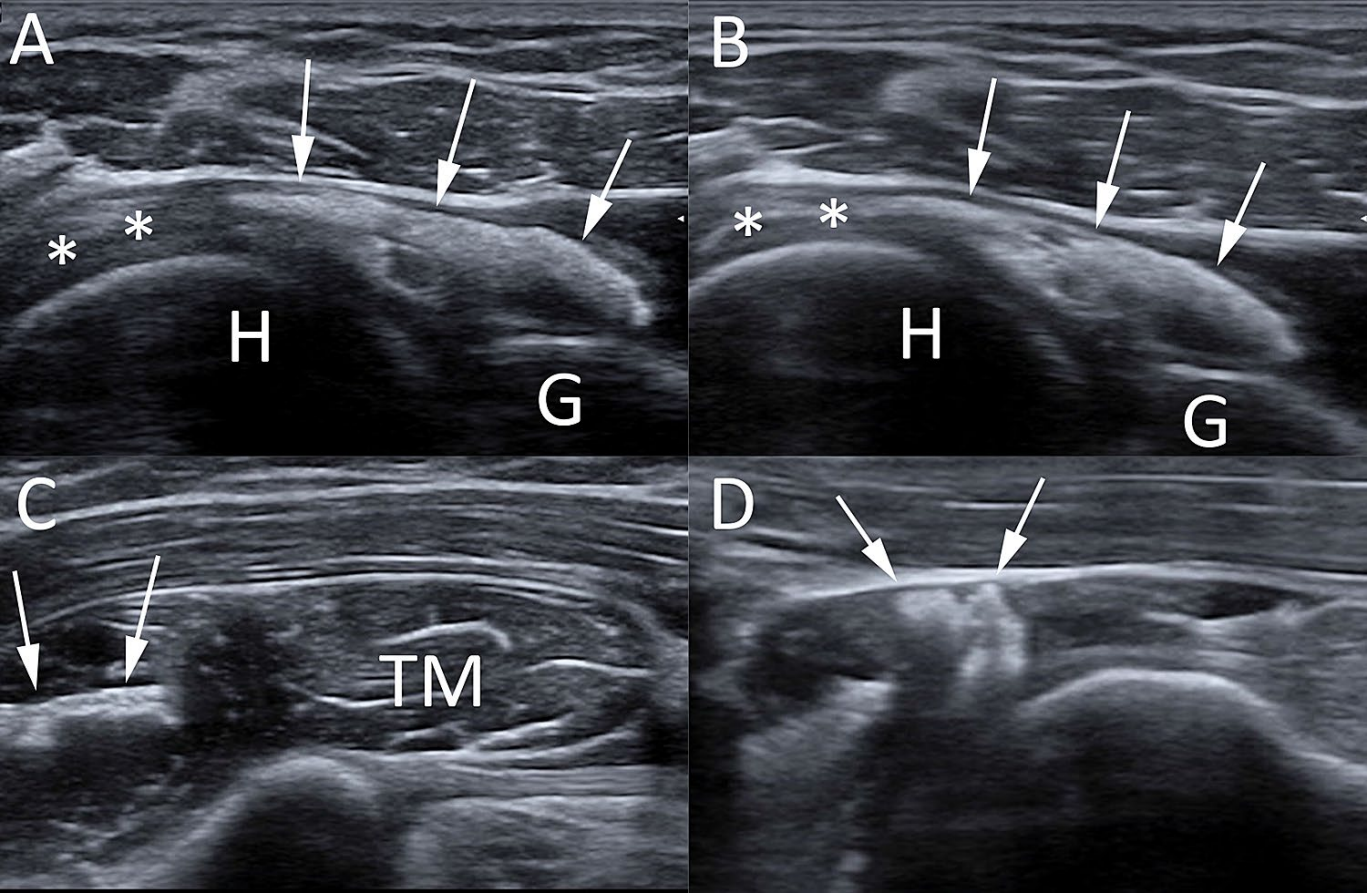
Intramuscular migration of RCCT in a 51-year-old female patient. Long-axis US images of the infraspinatus tendon (**A**, **B**) and short-axis US images of the infraspinatus fossa (**C**, **D**) show the intramuscular migration of calcific deposits with ill-defined margins (arrows) at the myotendineous junction of the infraspinatus tendon (asterisk). H = Humeral head; G = Glenoid; TM = Teres Minor muscle belly

Another unusual presentation of RCCT is represented by intramuscular migration of calcific deposits. Only few studies in literature reported this atypical presentation of RCCT. Generally, intramuscular migrations of calcific deposits occur at or close to the myotendineous junction of the supraspinatus and infraspinatus tendons (Fig. 9) [36]. It was postulated that, in the resorptive phase, calcific deposits grow in size and often migrate to the adjacent tissues as muscles [36, 62]. Pereira et al. reported coexisting intrasubstance delaminating-type tendon tear in their patients. They hypothesized that tears are created during migration and serve as intramuscular pathway for the calcification [36].

Migrated deposits generally have ill-defined contour and lower consistency than the stable portion of calcification they migrated from [62]. At MR, the migrated portion could demonstrate a higher signal intensity than the main stable calcium bulk, and a small neck, which has been referred to “comet-tail” appearance [44, 63]. Furthermore, the presence of calcific deposits within the muscle creates an inflammatory response that is easily identified with MR [36]. The substantial reactive edema, diffuse or isolated, in the muscle belly close to the calcific deposits may be confused with other entities as myotendineous junction injury, infectious processes or denervation edema of the involved muscles [36, 44]. Elements that allow to distinguish various entities are the slightly younger age group of the patients and the history of acute trauma in support of injury, the clinical history of infection for infectious process, and the uniform involvement of the entire affected muscle not associated with perimuscular fluid accumulation in denervation edema [36, 64, 65]. MR is the modality of choice to assess the diffuse edema observed in case of muscular extension of RCCT, but it is important to know patient’s clinical history and to perform CR or US first to identify the calcium deposits, in order to avoid a misinterpretation of MR images [36, 44].

### Atypical sites of calcific tendinopathy around the shoulder

In the shoulder, calcific tendinopathy typically affects the tendons of the rotator cuff and other sites are uncommon. To our knowledge, only few cases reported examples of calcific tendinopathy in the long head of biceps brachii tendon. The bicipital anchor and the distal myotendinous junction seem to be the two most vulnerable sites [66]. It has been described from case series and case reports as calcific opacities at plain radiographs or as inhomogeneous hyperechoic mass at US located along the anterior aspect of the proximal third of the biceps brachii muscle [66, 67]. Calcifications at the origin of long head of the biceps, such as biceps anchor or biceps labral complex, have been less described in literature and generally present an ovoid shape, with their position close to the upper glenoid being unchanged during external or internal rotation of the humerus [66, 68, 69]. However, it is unlikely these represent real calcific tendinopathy but rather calcific apposition over degenerated tendons.

**Fig. 10:**
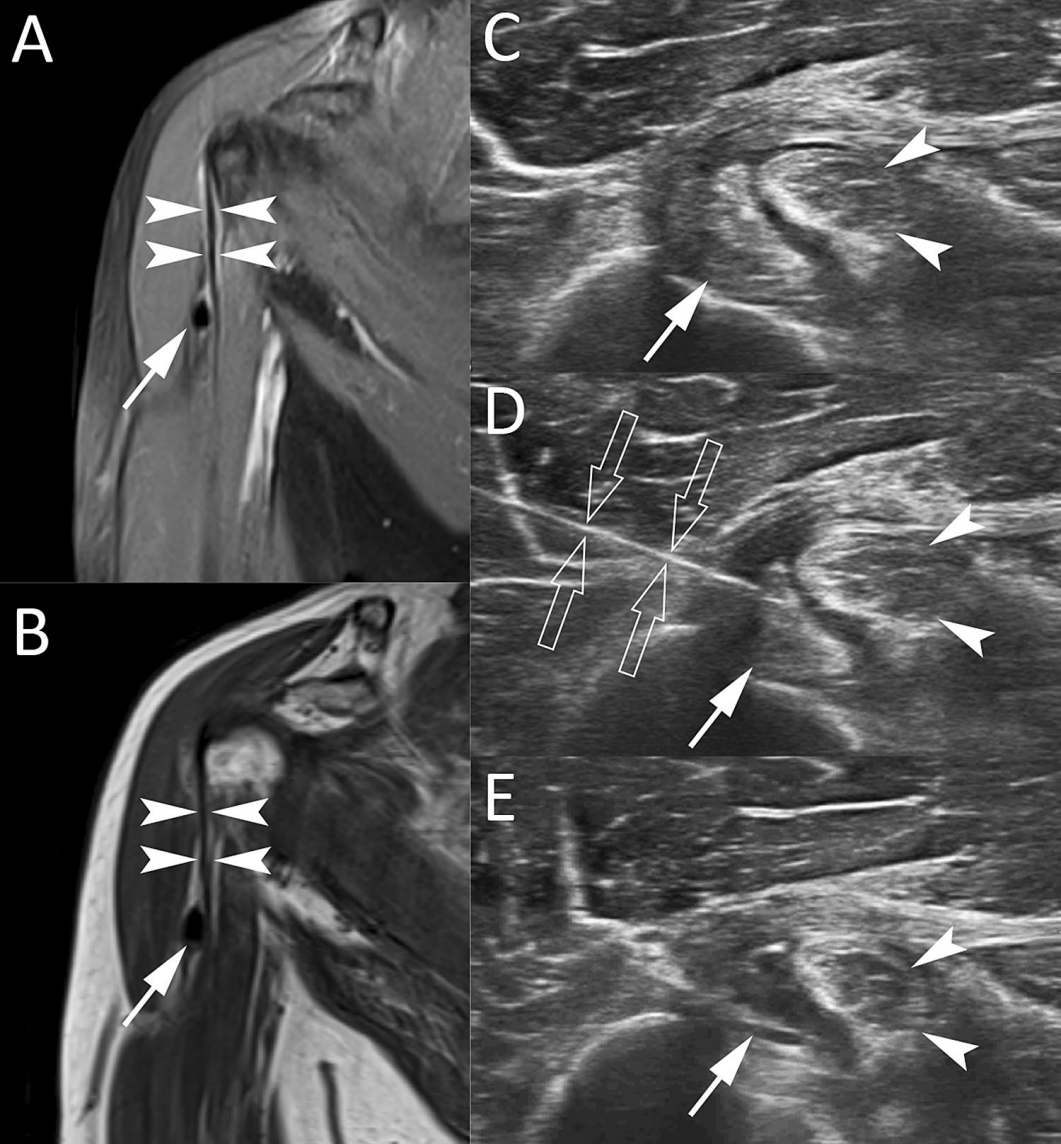
Calcific tendinopathy of the long head of the biceps brachii in a 54-year-old male patient. Coronal fat-suppressed proton-density weighted (**A**) and coronal T1-weighted (**B**) MR images show a hypointense calcification ovoid in shape (arrows) close to extra-articular portion of the long head of the biceps brachii (headarrows). Short-axis US images (**C**–**E**) demonstrate a soft calcification (arrows) within the sheath of the tendon (headarrows). Short-axis US image during the US-PICT (**D**) shows the needle (void arrows) inside the calcification, which presents anechoic content and a thin calcific wall at the end of the procedure (**E**)

Around the biceps, calcific tendinopathy can also occur within the synovial sheath of this tendon, in its vertical portion. These deposits usually have appearance similar to RCCT and a variable amount of fluid distension can be seen within the sheath itself. US can show the presence of a calcification with variable echogenicity, while MR is especially helpful when the calcific deposit is located in an unusual position (Fig. 10) [69].

**Fig. 11:**
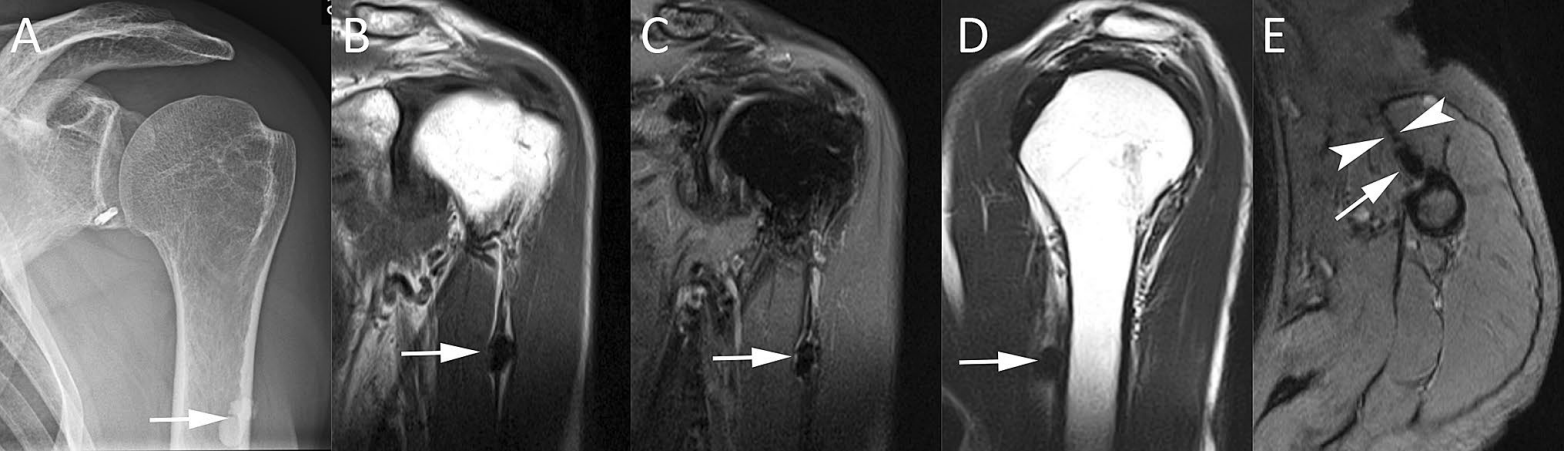
A 54-year-old female patient with left shoulder pain. Antero-posterior shoulder CR (**A**) displays a large calcification (arrow) close to the humeral shaft. Coronal T1-weighted (**B**), coronal fat-suppressed proton-density weighted (**C**), sagittal T2-weighted (**D**), and axial gradient-echo (**E**) MR images confirm the presence of a large calcification (arrows) at the insertion of pectoralis major tendon (arrowheads)

**Fig. 12:**
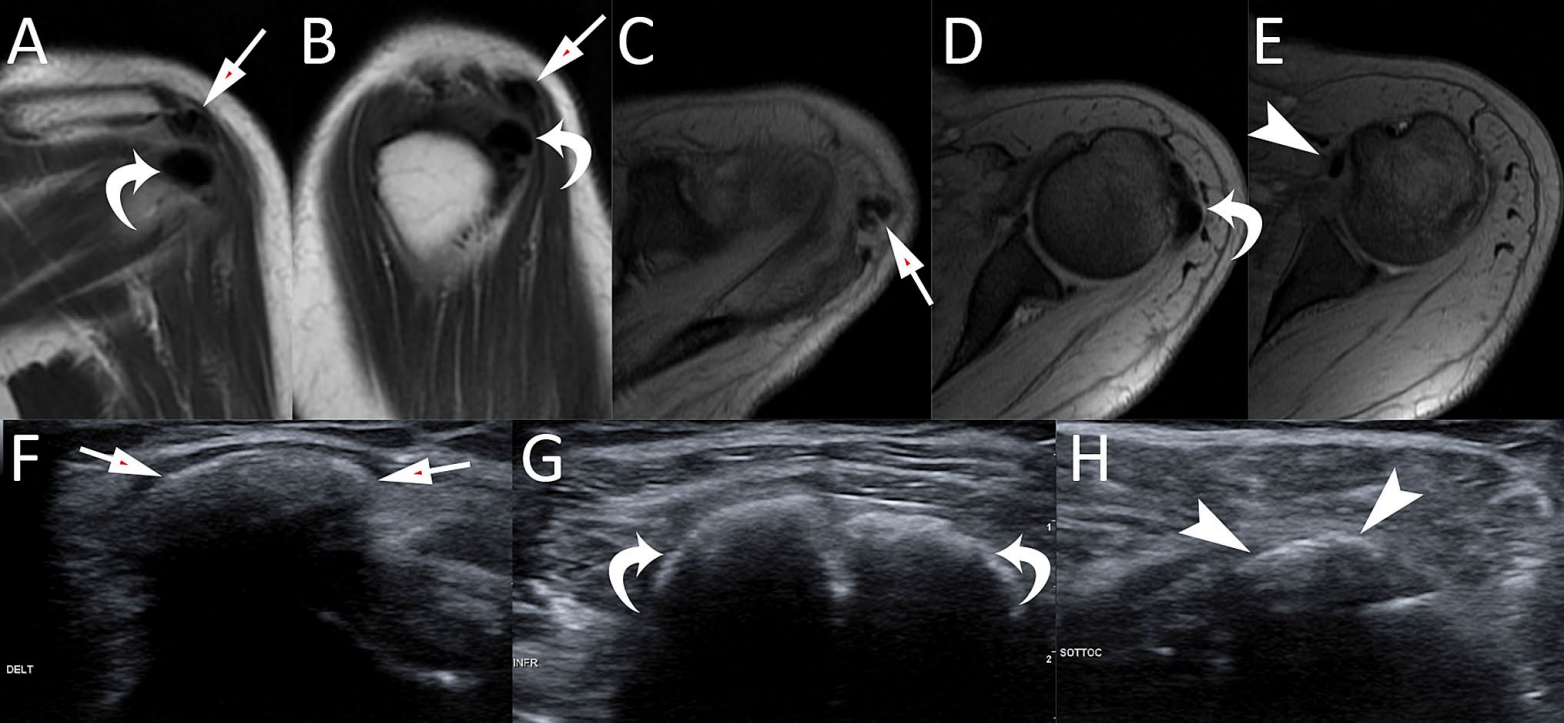
A 58-year-old female patient with intense left shoulder pain due to RCCT. Coronal proton-density weighted (**A**), sagittal proton-density weighted (**B**), axial gradient-echo (**C**, **D**, **E**) MR images show multiple calcifications located simultaneously in the deltoid (A, B, C, arrows), infraspinatus (**A**, **B**, **D**, curved arrows), and subscapularis (**E**) tendons. This rare picture was confirmed by US images showing hard calcifications in the deltoid (**F**, arrows), infraspinatus (**G**, curved arrows), and subscapularis (**H**, headarrows) tendons

Another atypical site of presentation of calcific tendinopathy is pectoralis major, again with few cases published on this condition (Fig. 11). In one of these case reports, the unusual presentation in the pectoralis tendon could lead to misdiagnosis of humeral chondroid neoplasm at imaging because of the bone marrow involvement and the cortical erosion showed by MR [70]. Calcific tendinopathy can be correctly diagnosed due to the presence of comet-tail or flame appearance of the calcification, described as a characteristic finding [39, 71, 72]. Another case report underlined that cortical erosion is particularly seen at areas of powerful traction, such as pectoralis major tendons, where the inflammation due to the mechanical effect of traction may result in bone resorption and tendon edema [73]. Although not previously reported, another rare site of presentation of calcific tendinopathy can be the deltoid (Fig. 12), as this condition can probably involve every tendinous structure [74].

## Conclusion

RCCT is a common and well-documented disease in the literature. Unusual presentations may lead to diagnostic challenge and mistakes that prolong the diagnostic pathway and delay the treatment. It is crucial for radiologists to recognize imaging findings of unusual presentations of RCCT and to differentiate it from other entities with the aim to prevent further unnecessary imaging examinations or interventional procedures.
